# Myopic Progression Associated with COVID-19 Pandemic in Korean Children with Myopia Using 0.01% Atropine Eyedrops

**DOI:** 10.3390/life16030407

**Published:** 2026-03-03

**Authors:** Dong Hyun Kim, Jihae Park, Jeong-Min Hwang, Hee Kyung Yang

**Affiliations:** 1 Department of Ophthalmology, Seoul National University College of Medicine, Seoul National University Bundang Hospital, Seongnam 13620, Republic of Korea; himrdh@gmail.com; 2 Department of Ophthalmology, Daegu Fatima Hospital, 99, Ayang-ro, Dong-gu, Daegu 41199, Republic of Korea; jihae.park85@gmail.com; 3 Department of Ophthalmology, Strabismus & Pediatric Ophthalmology Center, Kim’s Eye Hospital, 136, Yeongsin-ro, Yeongdeungpo-gu, Seoul 07301, Republic of Korea

**Keywords:** myopia, progression, Korean, children, COVID-19

## Abstract

**Background:** To evaluate the effect of the COVID-19 pandemic on myopic progression defined in terms of refractive change and axial length elongation in Korean children with myopia using 0.01% atropine eye drops. **Methods:** A retrospective review was performed on the medical records of 73 children aged 4 to 15 years with a baseline myopia of −0.50 diopters (D) or more who had used 0.01% atropine eye drops for more than 12 months before the onset of the COVID-19 pandemic in South Korea. The rate of myopic progression was compared between two periods: the pre- and post-pandemic eras, the latter of which was defined by the initiation of remote schooling in March 2020. At each visit, cycloplegic autorefraction and axial length were measured using a Zeiss IOL Master. Patients answered a questionnaire regarding their time spent on near work (computer, smartphone, reading, homework, after-school workbooks, drawing, etc.) and outdoors. **Results:** During the pandemic, in terms of refraction, myopia progressed at an average rate of −0.45 D/y, which was significantly faster than before the pandemic of −0.22D/y (*p* = 0.037). In contrast, axial length elongation was 0.22 mm/y and 0.19 mm/y before and after the pandemic, respectively, which was not significantly different (*p* = 0.546). Time spent on using computers, smartphones, and other near work significantly increased, while outdoor time had significantly decreased after the pandemic (paired *t*-test, all *p* < 0.001). The change in annual refractive myopic progression rate during the pandemic compared to the pre-pandemic period did not significantly correlate with changes in computer time, smartphone time, or other near work time (*p* = 0.134, 0.210, 0.863, respectively). However, the change in outdoor time showed a negative correlation with the change in annual myopic progression rate (r = −0.239, *p* = 0.041). **Conclusions:** Among Korean children aged 4 to 15 years receiving 0.01% atropine, the rate of myopic progression increased significantly in terms of refraction during the COVID-19 pandemic compared with the pre-pandemic period, whereas axial length progression did not change significantly.

## 1. Introduction

The prevalence of myopia is rising steadily around the world [1]. This trend poses a significant public health challenge due to the increasing likelihood of irreversible visual impairment associated with myopia [2]. In East Asian countries, such as South Korea, China, Japan, and Singapore, the prevalence of myopia among school-aged children is especially concerning [3,4]. There are marked differences in the prevalence of myopia among different populations and ethnic groups [5,6], with one of the highest being 96.5% reported in 19-year-old males in Korea [7]. This growing epidemic has been linked to genetic predisposition, as well as rapid environmental and behavioral changes associated with modernization, intense educational demands, and reduced outdoor exposure [8,9].

Outdoor activity has long been recognized as a protective factor against the onset and progression of myopia [10]. The mechanism is thought to involve exposure to higher illuminance levels and increased retinal dopamine release, which may inhibit the eyeball’s axial elongation [11,12]. In contrast, excessive near-work activities, such as reading, computer use, and smartphone viewing, have been associated with myopic shifts; however, evidence of their direct causal role remains inconclusive [10]. Thus, the balance between near work and outdoor activity represents a crucial environmental determinant of refractive development in children.

The outbreak of the global pandemic caused by the novel coronavirus (COVID-19) in late 2019 dramatically disrupted these environmental balances. To reduce the spread of the virus, governments worldwide implemented varying degrees of lockdowns and school closures [13]. In South Korea, strict lockdowns were avoided, but all schools transitioned to online education in March 2020 [14]. This transition altered the daily routines of millions of children, increasing time spent indoors and on digital screens while decreasing opportunities for outdoor play, sunlight exposure, and physical activity [15]. Studies from China, Israel, and other regions have demonstrated an increased prevalence of myopia and faster refractive shifts in children following pandemic-related home confinement [16,17,18]. Home quarantine caused a subsequent increase in near screen time for online learning, and limited outdoor activities. Zhou et al. [16] showed increased rates of myopia in Chinese school primary students of grade 2–4 during COVID-19. In South Korea, schools started online e-classes in March 2020 during the quarantine. Spending most of the time attending online e-classes watching digital devices inevitably led to increased near work and screen time, reduction in time spent outdoors as well as less sunlight exposure for school children. Shneor et al. [17] revealed that children became less active, less engaged in physical activity, and spent less time outdoors during COVID- 19. Reduced outdoor light exposure is important because of the role of light in the development of myopia and vitamin D. [19] Wong et al. [18] also reported increased near work as well as digital screen time, and limited outdoor activities during the COVID-19 pandemic.

The degree of lockdown associated with COVID-19 varied among different countries; for example, lockdown was strict by taking dynamic zero-COVID policy in China [20], whereas in South Korea, there were almost no lockdown restrictions and only online classes were conducted. Therefore, the impact of COVID-19 quarantine on myopic progression may exhibit regional variability. This contextual difference makes South Korea an ideal setting to assess the effect of moderate but sustained increases in near work without complete lockdown on myopia progression under pharmacologic control. This context raises important questions regarding the extent to which modest environmental disruptions, rather than strict confinement, influence myopic progression and how these changes interact with ongoing myopia control treatments in pediatric populations.

Low-dose atropine has become essential in the treatment of myopia [21]. Numerous clinical trials have demonstrated its effectiveness in slowing the progression of myopia and axial elongation in children [22,23]. However, its performance under adverse environmental conditions, such as during a pandemic when outdoor exposure decreases, has not been fully explored. There are several studies investigating the change in myopia progression among children during the COVID-19 pandemic [15,24]. However, social, ethnic and treatment differences such as the use of low dose atropine may differ among different study populations. Due to South Korea’s uniquely high educational intensity and digital infrastructure, this population offers valuable insights into the interaction between pharmacological and environmental factors in real-world settings. A critical unresolved issue is the degree to which environmental stressors—reduced outdoor activity and increased near-work demand—can diminish or even counteract the therapeutic effect of low-dose atropine. This highlights the need to evaluate pharmacologic therapy within the context of actual lifestyle patterns.

This study aimed to investigate the impact of the pandemic quarantine on myopic progression in children treated with 0.01% atropine eye drops at a tertiary hospital in South Korea, a setting with exceptionally high prevalence of myopia. Specifically, changes in refractive error and axial length were compared between the pre-pandemic and pandemic periods, along with their associations with time spent on near work and outdoor activities. Through this approach, the study sought not only to characterize pandemic-related myopic changes, but also to clarify how environmental changes interact with low-dose atropine treatment, offering deeper insight into the multifactorial nature of myopia progression.

## 2. Materials and Methods

### 2.1. Subjects

A retrospective review was conducted on the medical records on pediatric patients who had used 0.01% atropine eye drops for more than 12 months before the onset of the COVID-19 pandemic in South Korea. The rate of myopic progression was compared between two periods: the pre- and post-pandemic eras, the latter of which was defined by the initiation of remote schooling in March 2020. The study adhered to the principles of the Declaration of Helsinki and was approved by the Seoul National University Bundang Hospital Institutional Review Board (B-2508-989-102). Due to the retrospective design and anonymized data collection, written informed consent was waived.

Careful consideration was given to establish the inclusion criteria to ensure that the participants represented a homogeneous population of children with progressive myopia who were receiving consistent pharmacological treatment. Eligible children were between 4 and 15 years of age with a baseline refractive error of at least −0.50 diopters (D) in their more myopic eye. Participants with a documented myopia progression of at least 0.50 D in the year preceding the commencement of atropine treatment were included. Participants were required to have documented follow-up visits at least twice per year before and during the pandemic to allow for accurate annual comparisons [25].

Children were excluded if they had astigmatism greater than 1.50 D, anisometropia exceeding 1.00 D, or a best-corrected visual acuity worse than 20/25, or if they had a history of using other myopia control therapies, such as multifocal lenses or orthokeratology [26]. These criteria minimized the confounding effects of other refractive or pathological abnormalities. All participants received 0.01% atropine nightly in both eyes.

### 2.2. Ophthalmologic Examination

At each visit, cycloplegic autorefraction and axial length were measured. Cycloplegia was achieved using three drops of 1% cyclopentolate (Cyclogyl^®^, Alcon, Fort Worth, TX, USA), administered five minutes apart. Cycloplegic autorefraction was measured 30 min after the last drop using a Huvitz HRK-8000A autorefractor (Huvitz, Anyang-si, Gyeonggi-do, Republic of Korea) [27]. Five readings, all within 0.25 D of each other, were averaged. The spherical equivalent refractive error (SER) was calculated as the sphere plus half the cylindrical power [27]. Axial length was obtained using a Zeiss IOL Master (Carl Zeiss Meditec, Jena, Germany) [28]. Five readings, all of which were within 0.05 mm or less, were averaged [29].

### 2.3. Near Work Time and Outdoor Time Survey

To evaluate behavioral factors, a structured questionnaire that asked about how much time is spent each day on computers, smartphones, other close-up activities (reading, doing homework, working on after-school workbooks, drawing, etc.), and outdoor activities (playing sports, biking, swimming, having picnics, etc.) was handed out (Supplementary Material). Parents or guardians completed the survey. Participants were asked to recall their typical weekday and weekend routines before and after March 2020, which correspond to the pre- and pandemic periods, respectively. Average daily hours were computed by weighting weekday and weekend activity times.

### 2.4. Data Analyses

Statistical analyses were conducted using IBM SPSS Statistics for Windows, version 26.0 (IBM Corporation, Armonk, NY, USA). Continuous variables are expressed as mean ± standard deviation (SD). The Shapiro–Wilk test was used to verify the normality of data distribution. A paired *t*-test was used to compare the annualized rates of myopia progression, axial elongation, and lifestyle factors before and during the pandemic. Pearson correlation analysis was applied to examine the relationship between changes in refractive progression and changes in behavioral variables, such as near work and outdoor time. A *p*-value of less than 0.05 was considered statistically significant for all tests.

## 3. Results

### 3.1. Participant Characteristics

An initial retrospective review of 147 clinical records was conducted. Of these, 74 were excluded (31 for insufficient follow-up visits, 4 for other ocular diseases, 14 for poor treatment compliance, and 25 for concurrent myopia control treatments), resulting in a final cohort of 73 pediatric patients. Of those patients, 30 were male and 43 were female. The mean duration of treatment with 0.01% atropine was 35.8 ± 9.6 months (range, 21.9–65.3 months). At the start of treatment, the mean age was 9.9 ± 3.0 years (range, 4.0–15.6 years), the mean SER was −5.1 ± 1.9 diopters (range, −1.9 to −10.9 diopters), and the mean axial length was 25.1 ± 1.0 mm (range, 22.9–27.7 mm). At the onset of the quarantine period related to the COVID-19 pandemic, the mean age was 11.6 ± 2.8 years (range, 4.9–16.9 years), the mean SER was −5.5 ± 2.0 D (range, −1.8 to −10.5 D), and the mean axial length was 25.3 ± 1.0 mm (range, 23.2–27.8 mm). By the final visit, the mean age had increased to 12.6 ± 2.8 years (range, 6.0–18.0 years), the mean SER had increased to −6.0 ± 1.9 D (range, −1.8 to −11.0 D), and the mean axial length had increased to 25.5 ± 1.0 mm (range, 23.5–28.1 mm). These baseline and follow-up characteristics are summarized in Table 1.

### 3.2. Rate of Myopia Progression and Axial Length Elongation Before and After COVID-19

**Figure 1:**
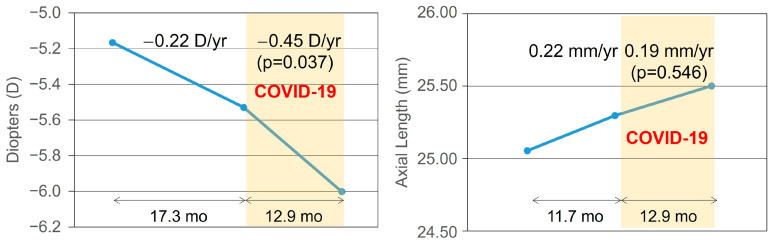
Annual rate of myopic SER progression and axial elongation before and after the COVID-19 pandemic among children using low dose atropine eye drops. The annual rate of myopic SER progression increased from −0.22 ± 0.79 diopters (D)/y before the pandemic to −0.45 ± 0.52 D/y during the pandemic, showing a significantly faster progression during the pandemic (*p* = 0.037). In contrast, the annual rate of axial elongation was 0.22 ± 0.44 mm and 0.19 ± 0.14 mm before and after the pandemic, showing no significant difference (*p* = 0.546).

Before the COVID-19 pandemic, the annual rate of myopic SER progression was −0.22 D per year. During the pandemic period, the annual rate of myopic SER progression was −0.45 D per year. The difference in annual myopic SER progression between the two periods was statistically significant (*p* = 0.037). Before the pandemic, the annual rate of axial length elongation was 0.22 mm per year. During the pandemic, the annual rate of axial length elongation was 0.19 mm per year. The difference in axial elongation between the pre-pandemic and pandemic periods was not statistically significant (*p* = 0.546). Individual values for refractive change and axial length change in each period are presented in Figure 1.

### 3.3. Near Work Time and Outdoor Time

**Figure 2:**
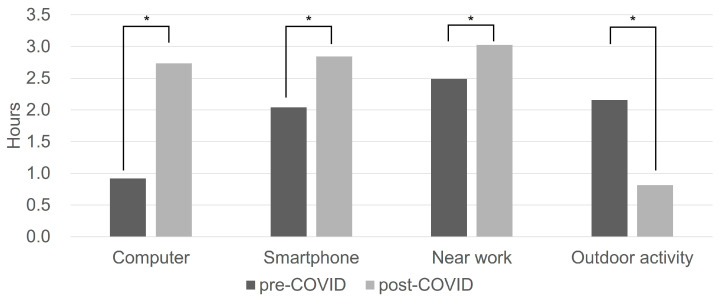
Changes in activity time before and after the COVID-19 pandemic. Computer and smartphone use, as well as other near work activities (reading, homework, after-school workbooks, drawing, etc.) all significantly increased after the pandemic. Meanwhile, time spent outdoors significantly decreased in all aspects (outdoor sports, biking, swimming, picnics in the park, etc.). (Paired *t*-test; asterisk indicates *p* < 0.001).

Before the pandemic, the mean daily duration of computer use was 0.9 ± 1.0 h, which increased to 2.7 ± 2.3 h during the pandemic. Smartphone use increased from 2.0 ± 1.6 h to 2.8 ± 2.3 h. Time spent on other near work, including reading, homework, after-school workbooks, and drawing, increased from 2.5 ± 1.8 h to 3.0 ± 2.0 h. In contrast, mean outdoor time decreased from 2.2 ± 1.8 h per day to 0.8 ± 0.9 h per day. All within-subject differences in activity time before and during the pandemic were statistically significant (paired *t*-test, all *p* < 0.001). These data are illustrated in Figure 2.

### 3.4. Correlation of Changes in Refractive Errors

**Figure 3:**
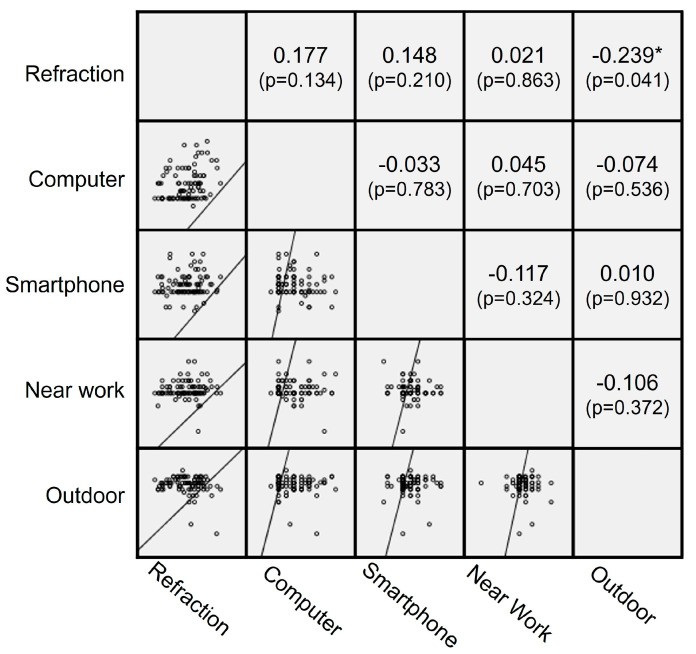
Correlation of changes in activity time with changes in the annual rate of myopic SER progression. Changes in time spent on computers, smartphones, and other near work were not significantly correlated with the changes in the annual rate of myopic SER progression (where a positive value indicates progression). However, there was a significant negative correlation between changes in annual rate of myopic SER progression and the change in time spent outdoors (r = −0.239, *p* = 0.041). The asterisk (*) indicates *p* < 0.05.

Correlation analyses were performed to evaluate the relationship between changes in behavioral variables and changes in the annual rate of myopia progression between the two periods. Changes in time spent on computers, smartphones, and other near work were not significantly correlated with the change in annual myopia progression rate (*p* = 0.134, 0.210, 0.863, respectively). In contrast, the change in outdoor time showed a negative correlation with the change in annual myopia progression rate (r = −0.239, *p* = 0.041). The scatterplots demonstrating these correlations are shown in Figure 3.

### 3.5. Subgroup Analysis by Age and Sex

Subgroup analyses were conducted to determine if the impact of the pandemic on myopia progression and lifestyle patterns varied by sex and age. Regarding sex, a significant increase in the annual rate of myopic SER progression was observed in males (pre-COVID: −0.04 D/year vs. post-COVID: −0.39 D/year; *p* = 0.038), whereas the change in females did not reach statistical significance (*p* = 0.167). However, axial length elongation showed no significant differences between the two periods for either sex (all *p* > 0.05). Both males and females reported drastically increased time spent on computers, smartphones and other near-work activities alongside substantially reduced outdoor activity (all *p* < 0.05). (Table 2) When analyzed by age group (<8, 8 ≤ Age <12, and ≥12 years), there were no statistically significant differences in SER progression or AL elongation within any age group between the pre- and post-COVID periods (all *p* > 0.05) (Table 3).

## 4. Discussion

Myopia has emerged as the most prevalent ocular disorder worldwide with its incidence rising steadily over the past decades. Although the onset and progression of myopia may differ across ethnic groups, the lifestyle disruption brought about by the COVID-19 pandemic has introduced a set of environmental pressures that may influence myopia progression on a global scale, especially in children. Understanding these environmental influences on myopia is crucial, as they can serve as both protective and risk-enhancing factors. In this study, the effect of the COVID-19 pandemic on myopic progression in Korean children receiving 0.01% atropine treatment was investigated. The results demonstrated that annual rate of myopic SER progression significantly increased during the pandemic compared to the pre-pandemic period, whereas axial length elongation remained relatively stable. This acceleration in refractive change coincided with a marked shift in daily habits, specifically increased digital device use and reduced outdoor activities. Notably, less time spent outdoors was significantly associated with faster myopia progression, while near-work activities did not show a similar correlation. These findings suggest that reduced outdoor exposure during the pandemic accelerated refractive change despite continuous low-dose atropine therapy. However, it is important to recognize that increased near-work demands and reduced time spent outdoors may be interrelated behavioral patterns rather than completely independent factors.

The rate of myopia has increased rapidly worldwide, especially in East Asia [7,30,31,32]. The Korean National Health and Nutrition Survey conducted from 2008 to 2011, which included 22,562 people aged ≥ 20 years, found that the prevalence of myopia (<−0.5 D) and high myopia (<−6.0 D) were 48.1% and 4.0%, respectively [33]. The prevalence of myopia sharply decreased from 78.9% in 20 to 29 years to 16.1% in 60 to 69 years of age. The risk factors of myopia were younger age and education level of university or higher, and shorter sunlight exposure time. The prevalence of myopia of 34.7% in Korean adults ≥ 40 years was comparable to that in other Asian countries in this population-based study, which show that the Korean younger generations are much more myopic than older generations. Notably, this survey was conducted before the COVID-19 pandemic, suggesting that current rates are likely even higher given the substantial lifestyle changes that followed. Regarding the risk factors for myopia, earlier epidemiologic studies showed that education, a possible reflection of cumulative engagement in near work activity, is a strong and consistent risk factor [7,34,35,36]. Later research identified that time spent outdoors, especially exposure to bright sunlight, as another critically important protective factor against myopia in children [8,9]. Therefore, the importance of outdoor activity could not be stressed enough for children in order to prevent myopic progression. Interestingly, current sunlight exposure time showed an inverse relationship with the myopia prevalence, even in adults with ceased eyeball elongation, along with body growth [33]. Sherwin et al. [37] observed higher levels of conjunctival ultraviolet autofluorescence (UVAF), a biomarker of outdoor light exposure, were associated with lower myopia prevalence, and males of younger age in Norfolk Islanders. This could reflect a kind of continuity in lifestyle behavior pattern. Individuals who spend more time outside as children tend to spend more time outside as adults, which may offer sustained protection from myopia [6,33].

The COVID-19 pandemic led to school closures and a shift to online classes, drastically altering children’s daily routines. Prolonged time spent indoors, combined with excessive use on digital devices, and reduced outdoor physical activities created conditions that may have contributed to accelerated myopia progression during the outbreak. The level of quarantine and school suspension policies during the COVID-19 pandemic varied across different regions worldwide. Wang et al. [38] found that the prevalence of myopia in school children aged 6–8 years became significantly greater after home confinement than before. Luo et al. [39] performed a meta-analysis of eight eligible studies, and they found a significant difference in SER especially in females as well as in urban areas, but no difference in axial length before and during the COVID-19 pandemic, which correlates with the results of this study. Along with environmental factors, race, and age, sex also plays an important role in myopia progression. Several studies have demonstrated meaningful sex-related differences in both prevalence and progression rates. Hyman et al. [40] reported that myopic girls showed faster refractive progression of 0.16 D than myopic boys over 3 years. Similarly, Fan et al. [41] and Zhou et al. [42] reported lower prevalence and less myopic progression among boys in Hong Kong and Chongqing, respectively. Behavioral factors likely contributed to these sex-related disparities. Girls have been shown to engage in longer durations of near work, screen time, and comparatively limited outdoor activity, all of which are established risk factors of myopia progression. Hormonal influences have also been proposed. Gong et al. [43] reported that a higher estrogen level in girls may also explain the sexual differences because estrogen may affect cell growth, signal transmission, as well as modification of eye tissues [44,45]. Such hormonal effects may interact with visual environmental stressors, amplifying the rate of refractive change. Meanwhile, the data indicates that the annual rate of myopic SER progression in boys accelerated significantly during the pandemic. This appears to be influenced by an increase in computer usage time (from approximately one to 3.5 h per day) and a concurrent decrease in outdoor activity. These results imply that boys may be more susceptible to drastic environmental changes.

**Figure 4:**
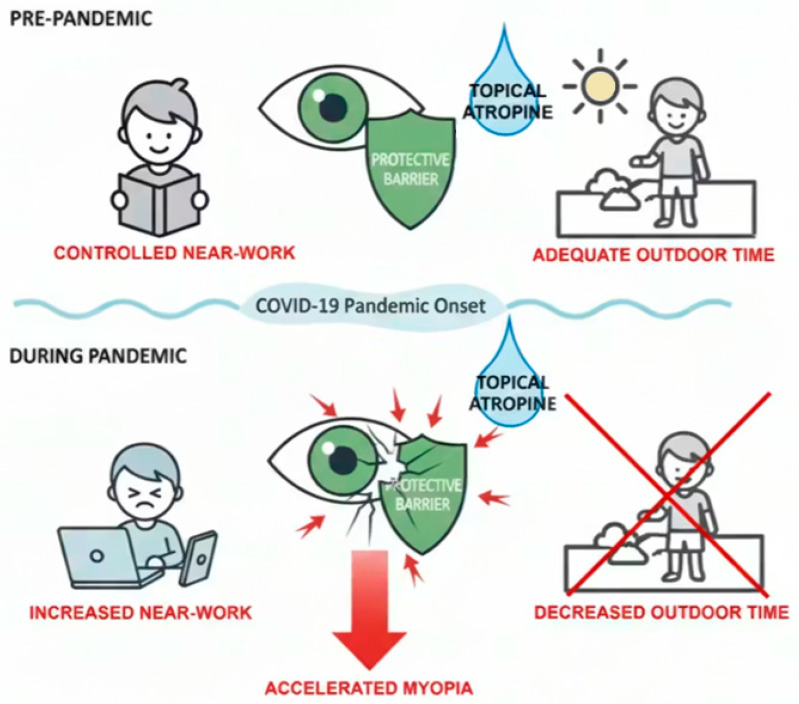
Summarization of myopia acceleration during COVID-19 pandemic.

Atropine, a nonselective muscarinic acetylcholine receptor antagonist, has been widely used to slow myopic progression in children [22]. Numerous studies have consistently demonstrated the efficacy of low-concentration atropine eye drops in controlling myopia [21,22,23]. A recent randomized, placebo-controlled low-concentration atropine for myopia progression (LAMP) study further confirmed this benefit, reporting reductions of 67%, 43%, and 27% in refractive progression and 51%, 29%, and 12% in axial elongation, over a period of 1 year with 0.05%, 0.025%, and 0.01% atropine, respectively [22]. The findings underscored a clear concentration-dependent effect, with 0.05% being the most effective in myopia control. However, its performance under adverse environmental conditions, such as those seen during the COVID-19 pandemic with markedly reduced outdoor exposure, remains insufficiently understood. In this study, Korean children aged 4 to 15 years old who received 0.01% atropine showed significantly faster rate of myopic SER progression during the pandemic, despite the absence of a significant change in axial length. This suggests that efficacy of 0.01% atropine may be insufficient to exert a meaningful effect under such conditions. Figure 4 provides a schematic summary of this concept, illustrating how increased near-work and reduced outdoor time during the pandemic may have overwhelmed the protective effect of low-dose atropine, potentially leading to accelerated myopia progression. Yum et al. [46] observed significant myopic progression after COVID-19 than before in those using 0.05% and 0.025% atropine in Korean children aged 5 to 10 years, but not in children aged 11 to 15 years. In contrast to the study by Yum et al., [46] which reported no significant difference in myopic progression before and after the COVID-19 pandemic in children treated with 0.01% atropine (−0.62 D/year vs. −0.67 D/year), this study demonstrated a significant increase in myopia progression rate from −0.22 D/year to −0.45 D/year while receiving 0.01% atropine. Several factors may explain this discrepancy. First, the 0.01% atropine group in Yum et al. [46] was relatively small (n = 15) and older (mean age 11.7 years) than the younger cohorts that generally exhibit more rapid myopic progression and stronger responses to environmental changes. In contrast, the present study included a larger sample size (n = 73) with a younger mean age (9.9 years), a population potentially more susceptible to both atropine treatment and environmental factors. The younger age groups in Yum et al.’s [46] study (5–7 and 8–10 years), who were not predominantly represented in their 0.01% atropine group, showed the largest pandemic-related myopia progression, reinforcing the age-dependent susceptibility observed in the present findings. Second, there were differences in measurement protocol (non-cycloplegic [46] vs. cycloplegic refraction). Moreover, participants in the present study had a longer cumulative duration of atropine use and were monitored over an extended period, which may have allowed the pandemic-related environmental impact to manifest more and increased sensitivity to subtle refractive changes that might otherwise remain undetected.

In this study, a significant increase was observed in the rate of SER progression during the pandemic, whereas axial length elongation did not show a corresponding significant change. This finding is consistent with previous reports suggesting that low-dose atropine can mitigate refractive changes without significantly affecting axial growth [47,48]. This discrepancy between refractive and axial length changes may be attributed to several factors. First, environmental stressors during the pandemic might have primarily influenced the accommodative state rather than immediate axial elongation [49,50]. Second, it is difficult to detect statistical significance in axial length changes because the annual changes are small (typically 0.2–0.3 mm). Thus, limited sample size or short follow-up periods may fail to capture these structural differences [51]. Lastly, short-term physiological rearrangements, such as atropine-induced changes in choroidal thickness, may influence refractive values before net axial growth is affected [52]. These results highlight the need for further research into the complex relationship between refractive and biometric myopia parameters.

This study has several limitations. Firstly, patients with a wide range of myopia, including those with high myopia up to −10.00 D, were included in the analysis. Secondly, other risk factors such as parental myopia were not evaluated in this study. Additional data may be necessary to evaluate the associated risk factors of myopia progression. Thirdly, the data regarding time spent on various activities may be subject to bias in parental recall, which could affect the precision of the reported environmental influences. Lastly, children who were not treated with atropine eye drops were not included since they were generally not referred to tertiary hospitals. A direct comparison between the atropine-treated and untreated children before and during the COVID-19 pandemic would have provided a clearer insight into the relative protective effect of atropine under environmental stress, such as increased near work and reduced outdoor exposure on myopia progression. Further prospective studies including untreated controls and various atropine concentrations are needed to determine the most effective atropine dosage or combination strategies with other myopia control treatments, such as optical interventions. This is particularly important in conditions under increased near work and reduced outdoor exposure, where treatment response may differ from previous studies. In parallel, the interaction between these environmental factors and atropine efficiency requires deeper investigation, as understanding how varying concentrations perform within contemporary lifestyle patterns will be essential for guiding more effective treatment decisions in this emerging “new normal.”

## 5. Conclusions

In conclusion, this study demonstrated a significantly higher rate of myopic SER progression in Korean children during the COVID-19 pandemic compared with the pre-pandemic period. Notably, this acceleration was observed even with ongoing 0.01% atropine treatment, which may suggest that very low-dose atropine may not be sufficient to counteract heightened environmental stressors. It is important to recognize that many of the behavioral and educational changes introduced during the pandemic have persisted. Many schools continue to rely heavily on digital device-based learning methods, and online or hybrid formats have become integrated into routine educational practice. These shifts have led to sustained high near-work demands and a continued reduction in outdoor exposure in children’s daily lives. As a result, the environmental conditions that likely contributed to accelerated myopia progression during the pandemic may still be present today. In summary, the findings of this study remain highly relevant and may offer valuable insights for understanding myopia progression within the current educational and lifestyle context in South Korea, even after the pandemic has officially ended. Its results also emphasize the importance of considering persistent environmental risk factors and continuously re-evaluating the appropriate treatment, even in children already receiving low-dose atropine eye drops.

For future public health crises, these findings suggest that pharmacological treatment alone may not suffice. It should be integrated with institutional policies that encourage outdoor activity and limit digital strain. For clinicians, the evidence highlights the need for a more proactive approach, including intensifying treatment when a child’s lifestyle undergoes substantial change. Overall, this study emphasizes the shared responsibility of healthcare and educational authorities in protecting children’s visual health.

## Figures and Tables

**Table 1:** Patients’ characteristics.

Variables	
Sex (male:female)	30:43
Duration of Atropine Use (months)	35.8 ± 9.6 (21.9–65.3)
Age (years)	
Baseline	9.9 ± 3.0 (4.0–15.6)
Quarantine onset	11.6 ± 2.8 (4.9–16.9)
Final	12.6 ± 2.8 (6.0–18.0)
SER (Diopters)	
Baseline	−5.1 ± 1.9 (−1.9–10.9)
Quarantine onset	−5.5 ± 2.0 (−1.8–10.5)
Final	−6.0 ± 1.9 (−1.8–11.0)
Axial length (mm)	
Baseline	25.1 ± 1.0 (22.9–27.7)
Quarantine onset	25.3 ± 1.0 (23.2–27.8)
Final	25.5 ± 1.0 (23.5–28.1)

Mean ± SD (range, minimum–maximum).

**Table 2:** Subgroup analysis by sex.

	Pre COVID	Post COVID	*p* Value
SER (Diopters)			
Male (*n* = 30)	−0.04 (0.26 to −0.40)	−0.39 (−0.06 to −0.73)	0.038
Female (*n* = 43)	−0.26 (0.07 to −0.63)	−0.39 (−0.05 to −0.98)	0.167
Axial length (mm)			
Male	−0.12 (0.04 to 0.29)	0.16 (0.09 to 0.31)	0.117
Female	0.13 (0.03 to 0.28)	0.16 (0.07 to 0.29)	0.435
Computer (hour)			
Male	1.0 (0.0 to 2.0)	3.5 (1.4 to 5.3)	<0.001
Female	0.5 (0.0 to 1.0)	2.0 (1.0 to 3.0)	<0.001
Smartphone (hour)			
Male	1.8 (1.0 to 3.0)	2.0 (1.0 to 4.0)	0.015
Female	2.0 (1.0 to 3.0)	2.0 (1.0 to 5.0)	0.001
Near Work (hour)			
Male	2.0 (1.0 to 4.0)	2.0 (1.8 to 4.0)	0.020
Female	2.0 (1.0 to 4.0)	2.0 (2.0 to 4.0)	0.003
Outdoor Activity (hour)			
Male	2.0 (1.0 to 4.0)	0.5 (0.1 to 1.0)	<0.001
Female	2.0 (1.0 to 3.0)	1.0 (0.5 to 1.0)	<0.001

*p* value by Wilcoxon signed rank sum test, Median (Interquartile range).

**Table 3:** Subgroup analysis by age.

	Pre-COVID-19	Post-COVID-19	*p* Value
SER (Diopters)			
<8 years (*n* = 12)	−0.36 (0.26 to −1.26)	−0.78 (−0.14 to −1.26)	0.440
8 ≤ Age < 12 (*n* = 41)	−0.25 (0.03 to −0.64)	−0.42 (−0.05 to −0.84)	0.070
≥12 years (*n* = 20)	−0.14 (0.23 to −0.34)	−0.21 (0.00 to −0.50)	0.256
Axial length (mm)			
<8 years	0.29 (0.14 to 0.48)	0.26 (0.18 to 0.36)	0.440
8 ≤ Age < 12	0.14 (0.03 to 0.26)	0.16 (0.09 to 0.30)	0.516
≥12 years	0.07 (0.02 to 0.15)	0.11 (0.05 to 0.19)	0.095

*p* value by Wilcoxon signed rank sum test, Median (Interquartile range).

## Data Availability

The raw data supporting the conclusions of this article will be made available by the authors on request. For data requests, please contact 98614@snubh.org.

## Supplementary Materials

The following supporting information can be downloaded at https://www.mdpi.com/article/10.3390/life16030407/s1, Table S1: Questionnaire on Changes in Near Work and Outdoor Activity Time (Korean and English).

## References

[1] Liang J., Pu Y., Chen J., Liu M., Ouyang B., Jin Z., Ge W., Wu Z., Yang X., Qin C. (2025). Global prevalence, trend and projection of myopia in children and adolescents from 1990 to 2050: A comprehensive systematic review and meta-analysis. Br. J. Ophthalmol..

[2] Flitcroft D.I. (2012). The complex interactions of retinal, optical and environmental factors in myopia aetiology. Prog. Retin. Eye Res..

[3] Han X., Liu C., Chen Y., He M. (2022). Myopia prediction: A systematic review. Eye.

[4] Grzybowski A., Kanclerz P., Tsubota K., Lanca C., Saw S.M. (2020). A review on the epidemiology of myopia in school children worldwide. BMC Ophthalmol..

[5] Vitale S., Sperduto R.D., Ferris F.L. (2009). Increased prevalence of myopia in the United States between 1971–1972 and 1999–2004. Arch. Ophthalmol..

[6] Kempen J.H., Mitchell P., Lee K.E., Tielsch J.M., Broman A.T., Taylor H.R., Ikram M.K., Congdon N.G., O’Colmain B.J., Eye Diseases Prevalence Research G. (2004). The prevalence of refractive errors among adults in the United States, Western Europe, and Australia. Arch. Ophthalmol..

[7] Jung S.K., Lee J.H., Kakizaki H., Jee D. (2012). Prevalence of myopia and its association with body stature and educational level in 19-year-old male conscripts in seoul, South Korea. Investig. Ophthalmol. Vis. Sci..

[8] Rose K.A., Morgan I.G., Ip J., Kifley A., Huynh S., Smith W., Mitchell P. (2008). Outdoor activity reduces the prevalence of myopia in children. Ophthalmology.

[9] Dirani M., Tong L., Gazzard G., Zhang X., Chia A., Young T.L., Rose K.A., Mitchell P., Saw S.M. (2009). Outdoor activity and myopia in Singapore teenage children. Br. J. Ophthalmol..

[10] Wu P.C., Tsai C.L., Wu H.L., Yang Y.H., Kuo H.K. (2013). Outdoor activity during class recess reduces myopia onset and progression in school children. Ophthalmology.

[11] French A.N., Ashby R.S., Morgan I.G., Rose K.A. (2013). Time outdoors and the prevention of myopia. Exp. Eye Res..

[12] Kim D.H., Hwang J.M., Yang H.K. (2025). Topical Dopamine Application on Form-Deprivation Myopia in Rabbits. Life.

[13] UNESCO COVID-19 Educational Disruption and Response. UNESCO, 24 March 2020. https://www.unesco.org/en/articles/covid-19-educational-disruption-and-response.

[14] Kim D.H., Lee H.J., Lin Y., Kang Y.J. (2021). Changes in academic performance in the online, integrated system-based curriculum implemented due to the COVID-19 pandemic in a medical school in Korea. J. Educ. Eval. Health Prof..

[15] Laan D., Tan E.T.C., Huis In Het Veld P.I., Jellema H.M., Jenniskens K. (2024). Myopia progression in children during home confinement in the COVID-19 pandemic: A systematic review and meta-analysis. J. Optom..

[16] Zhou W., Li Q., Chen H., Liao Y., Wang W., Pei Y., Li S., Zhang W., Wang Q., Wang X. (2022). Trends of myopia development among primary and junior school students in the post-COVID-19 epidemic period. Front. Public Health.

[17] Shneor E., Doron R., Levine J., Zimmerman D.R., Benoit J.S., Ostrin L.A., Gordon-Shaag A. (2021). Objective Behavioral Measures in Children before, during, and after the COVID-19 Lockdown in Israel. Int. J. Environ. Res. Public Health.

[18] Wong C.W., Tsai A., Jonas J.B., Ohno-Matsui K., Chen J., Ang M., Ting D.S.W. (2021). Digital Screen Time During the COVID-19 Pandemic: Risk for a Further Myopia Boom?. Am. J. Ophthalmol..

[19] Guggenheim J.A., Williams C., Northstone K., Howe L.D., Tilling K., St Pourcain B., McMahon G., Lawlor D.A. (2014). Does vitamin D mediate the protective effects of time outdoors on myopia? Findings from a prospective birth cohort. Investig. Ophthalmol. Vis. Sci..

[20] Ba Z., Li Y., Ma J., Qin Y., Tian J., Meng Y., Yi J., Zhang Y., Chen F. (2023). Reflections on the dynamic zero-COVID policy in China. Prev. Med. Rep..

[21] Chia A., Lu Q.S., Tan D. (2016). Five-Year Clinical Trial on Atropine for the Treatment of Myopia 2: Myopia Control with Atropine 0.01% Eyedrops. Ophthalmology.

[22] Yam J.C., Jiang Y., Tang S.M., Law A.K.P., Chan J.J., Wong E., Ko S.T., Young A.L., Tham C.C., Chen L.J. (2019). Low-Concentration Atropine for Myopia Progression (LAMP) Study: A Randomized, Double-Blinded, Placebo-Controlled Trial of 0.05%, 0.025%, and 0.01% Atropine Eye Drops in Myopia Control. Ophthalmology.

[23] Navarra P., Buzzonetti L., Amico V., Cro M., Federico B. (2025). A systematic review with meta-analysis on the efficacy of 0.01% atropine eyedrops in preventing myopia progression in worldwide children’s populations. Front. Pharmacol..

[24] Zhang X.J., Zhang Y., Kam K.W., Tang F., Li Y., Ng M.P.H., Young A.L., Ip P., Tham C.C., Chen L.J. (2023). Prevalence of Myopia in Children Before, During, and After COVID-19 Restrictions in Hong Kong. JAMA Netw. Open.

[25] Flitcroft D.I., He M., Jonas J.B., Jong M., Naidoo K., Ohno-Matsui K., Rahi J., Resnikoff S., Vitale S., Yannuzzi L. (2019). IMI-Defining and Classifying Myopia: A Proposed Set of Standards for Clinical and Epidemiologic Studies. Investig. Ophthalmol. Vis. Sci..

[26] Bao J., Yang A., Huang Y., Li X., Pan Y., Ding C., Lim E.W., Zheng J., Spiegel D.P., Drobe B. (2022). One-year myopia control efficacy of spectacle lenses with aspherical lenslets. Br. J. Ophthalmol..

[27] Pedersen H.R., Svarverud E., Hagen L.A., Gilson S.J., Baraas R.C. (2023). Comparing ocular biometry and autorefraction measurements from the Myopia Master with the IOLMaster 700 and the Huvitz HRK-8000A autorefractor. Ophthalmic Physiol. Opt..

[28] Jiang J., Pan X., Zhou M., Wang X., Zhu H., Li D. (2022). A comparison of IOLMaster 500 and IOLMaster 700 in the measurement of ocular biometric parameters in cataract patients. Sci. Rep..

[29] Kimura S., Hasebe S., Miyata M., Hamasaki I., Ohtsuki H. (2007). Axial length measurement using partial coherence interferometry in myopic children: Repeatability of the measurement and comparison with refractive components. Jpn. J. Ophthalmol..

[30] He M., Zeng J., Liu Y., Xu J., Pokharel G.P., Ellwein L.B. (2004). Refractive error and visual impairment in urban children in southern china. Investig. Ophthalmol. Vis. Sci..

[31] Sawada A., Tomidokoro A., Araie M., Iwase A., Yamamoto T., Tajimi Study G. (2008). Refractive errors in an elderly Japanese population: The Tajimi study. Ophthalmology.

[32] Lin L.L., Shih Y.F., Hsiao C.K., Chen C.J. (2004). Prevalence of myopia in Taiwanese schoolchildren: 1983 to 2000. Ann. Acad. Med. Singap..

[33] Kim E.C., Morgan I.G., Kakizaki H., Kang S., Jee D. (2013). Prevalence and risk factors for refractive errors: Korean National Health and Nutrition Examination Survey 2008–2011. PLoS ONE.

[34] Wu H.M., Seet B., Yap E.P., Saw S.M., Lim T.H., Chia K.S. (2001). Does education explain ethnic differences in myopia prevalence? A population-based study of young adult males in Singapore. Optom. Vis. Sci..

[35] Richler A., Bear J.C. (1980). Refraction, nearwork and education. A population study in Newfoundland. Acta Ophthalmol..

[36] Saw S.M., Cheng A., Fong A., Gazzard G., Tan D.T., Morgan I. (2007). School grades and myopia. Ophthalmic Physiol. Opt..

[37] Sherwin J.C., Hewitt A.W., Coroneo M.T., Kearns L.S., Griffiths L.R., Mackey D.A. (2012). The association between time spent outdoors and myopia using a novel biomarker of outdoor light exposure. Investig. Ophthalmol. Vis. Sci..

[38] Wang J., Li Y., Musch D.C., Wei N., Qi X., Ding G., Li X., Li J., Song L., Zhang Y. (2021). Progression of Myopia in School-Aged Children After COVID-19 Home Confinement. JAMA Ophthalmol..

[39] Luo Z., Guo C., Yang X., Zhang M. (2023). Comparison of myopia progression among Chinese schoolchildren before and during COVID-19 pandemic: A meta-analysis. Int. Ophthalmol..

[40] Hyman L., Gwiazda J., Hussein M., Norton T.T., Wang Y., Marsh-Tootle W., Everett D. (2005). Relationship of age, sex, and ethnicity with myopia progression and axial elongation in the correction of myopia evaluation trial. Arch. Ophthalmol..

[41] Fan D.S., Lam D.S., Lam R.F., Lau J.T., Chong K.S., Cheung E.Y., Lai R.Y., Chew S.J. (2004). Prevalence, incidence, and progression of myopia of school children in Hong Kong. Investig. Ophthalmol. Vis. Sci..

[42] Zhou W.J., Zhang Y.Y., Li H., Wu Y.F., Xu J., Lv S., Li G., Liu S.C., Song S.F. (2016). Five-Year Progression of Refractive Errors and Incidence of Myopia in School-Aged Children in Western China. J. Epidemiol..

[43] Gong J.F., Xie H.L., Mao X.J., Zhu X.B., Xie Z.K., Yang H.H., Gao Y., Jin X.F., Pan Y., Zhou F. (2015). Relevant factors of estrogen changes of myopia in adolescent females. Chin. Med. J..

[44] Xie H., Mao X., Yang H., Xie Z., Pan Y., Gao Y. (2014). Analysis on the relationship between adolescent myopia and serum sex hormone. Zhonghua Yi Xue Za Zhi.

[45] Kobayashi K., Mandai M., Suzuma I., Kobayashi H., Okinami S. (2002). Expression of estrogen receptor in the choroidal neovascular membranes in highly myopic eyes. Retina.

[46] Yum H.R., Park S.H., Shin S.Y. (2021). Influence of coronavirus disease 2019 on myopic progression in children treated with low-concentration atropine. PLoS ONE.

[47] Repka M.X., Weise K.K., Chandler D.L., Wu R., Melia B.M., Manny R.E., Kehler L.A.F., Jordan C.O., Raghuram A., Summers A.I. (2023). Low-Dose 0.01% Atropine Eye Drops vs Placebo for Myopia Control: A Randomized Clinical Trial. JAMA Ophthalmol..

[48] Yam J.C., Khanal S., Phillips J.R. (2025). Does 0.01% atropine have a place as a myopia control therapy?. Ophthalmic Physiol. Opt..

[49] Mohan A., Sen P., Shah C., Datt K., Jain E. (2021). Binocular Accommodation and Vergence Dysfunction in Children Attending Online Classes During the COVID-19 Pandemic: Digital Eye Strain in Kids (DESK) Study-2. J. Pediatr. Ophthalmol. Strabismus.

[50] Pena-Verdeal H., Noya-Padin V., Garcia-Queiruga J., Nores-Palmas N., Giraldez M.J., Yebra-Pimentel E. (2024). Temporal Variations in Convergence Insufficiency Symptomatic Status among University Students before and after COVID-19: A Longitudinal Analysis from 2018 to 2023. Life.

[51] Fu A., Stapleton F., Wei L., Wang W., Zhao B., Watt K., Ji N., Lyu Y. (2020). Effect of low-dose atropine on myopia progression, pupil diameter and accommodative amplitude: Low-dose atropine and myopia progression. Br. J. Ophthalmol..

[52] Chiang S.T., Turnbull P.R.K., Phillips J.R. (2020). Additive effect of atropine eye drops and short-term retinal defocus on choroidal thickness in children with myopia. Sci. Rep..

