# Supplementary material for: Myopic Progression Associated with COVID-19 Pandemic in Korean Children with Myopia Using 0.01% Atropine Eyedrops
Source: Life (Basel). 2026 Mar 3;16(3):407. doi: 10.3390/life16030407 (PMC13027977; doi:10.3390/life16030407)
Supplement: Supplementary file 1 [file life-16-00407-s001.zip › life-4075304-supplementary.pdf]

설문항목

■ 주중 하루 평균 컴퓨터 사용시간은 얼마입니까?

☐ 2019년(코로나 전) 평균______시간 ☐ 2020년(코로나 후) 평균______시간

■ 주말 하루 평균 컴퓨터 사용시간은 얼마입니까?

☐ 2019년(코로나 전) 평균______시간 ☐ 2020년(코로나 후) 평균______시간

■ 주중 하루 평균 스마트폰 사용시간은 얼마입니까?

☐ 2019년(코로나 전) 평균______시간 ☐ 2020년(코로나 후) 평균______시간

■ 주말 하루 평균 스마트폰 사용시간은 얼마입니까?

☐ 2019년(코로나 전) 평균______시간 ☐ 2020년(코로나 후) 평균______시간

■ 기타 근거리 작업(독서, 숙제, 학습지, 미술, 악기연주 등)은 주중 얼마입니까?

☐ 2019년(코로나 전) 평균______시간 ☐ 2020년(코로나 후) 평균______시간

■ 기타 근거리 작업(독서, 숙제, 학습지, 미술, 악기연주 등) 은 주말 얼마입니까?

☐ 2019년(코로나 전) 평균______시간 ☐ 2020년(코로나 후) 평균______시간

■ 주중 하루 평균 야외활동 시간은 얼마입니까?

☐ 2019년(코로나 전) 평균______시간 ☐ 2020년(코로나 후) 평균______시간

■ 주말 하루 평균 야외활동 시간은 얼마입니까?

☐ 2019년(코로나 전) 평균______시간 ☐ 2020년(코로나 후) 평균______시간

Survey Items

■ What was your average daily computer usage time on weekdays?

☐ 2019 (before COVID-19): _____ hours ☐ 2020 (after COVID-19): _____ hours

■ What was your average daily computer usage time on weekends?

☐ 2019 (before COVID-19): _____ hours ☐ 2020 (after COVID-19): _____ hours

■ What was your average daily smartphone usage time on weekdays?

☐ 2019 (before COVID-19): _____ hours ☐ 2020 (after COVID-19): _____ hours

■ What was your average daily smartphone usage time on weekends?

☐ 2019 (before COVID-19): _____ hours ☐ 2020 (after COVID-19): _____ hours

■ How much time did you spend on other near-work activities (e.g., reading, homework, worksheets, art, musical instruments) on weekdays?

☐ 2019 (before COVID-19): _____ hours ☐ 2020 (after COVID-19): _____ hours

■ How much time did you spend on other near-work activities (e.g., reading, homework, worksheets, art, musical instruments) on weekends?

☐ 2019 (before COVID-19): _____ hours ☐ 2020 (after COVID-19): _____ hours

■ What was your average daily outdoor activity time on weekdays?

☐ 2019 (before COVID-19): _____ hours ☐ 2020 (after COVID-19): _____ hours

■ What was your average daily outdoor activity time on weekends?

☐ 2019 (before COVID-19): _____ hours ☐ 2020 (after COVID-19): _____ hours
